# Editorial: Quality of Ornamental Crops: Effect of Genotype, Preharvest, and Improved Production Chains on Quality Attributes of Ornamental Crops

**DOI:** 10.3389/fpls.2022.918864

**Published:** 2022-08-03

**Authors:** Julian C. Verdonk, Antonio Ferrante, Margherita Irene Beruto, Peter Batt, Renato Paiva, Rob E. Schouten, Patricia Duarte de Oliveira Paiva

**Affiliations:** ^1^Horticulture and Product Physiology, Plant Science Group, Wageningen University and Research, Wageningen, Netherlands; ^2^University of Milan, Milan, Italy; ^3^Istituto Regionale per la Floricoltura, Sanremo, Italy; ^4^Curtin University, Perth, WA, Australia; ^5^Departamento de Biologia, Instituto de Ciências Biológicas, Universidade Federal de Lavras, Lavras, Brazil; ^6^Food and Bio Based Research, Wageningen University and Research, Wageningen, Netherlands; ^7^Departamento de Agricultura, Escola de Ciências Agrárias, Universidade Federal de Lavras, Lavras, Brazil

**Keywords:** ornamental plant, postharvest, biotechnology, plant physiology, floriculture

Acceptance of ornamental crops depends on the aesthetic value, the combination of flower number, shape, and size, as well as on the color, fragrance, and uniformity of blooming of these flowers. Plant shape, color, and patterning also are important. Vase life and keeping quality are often dependent on these features remaining at certain values, and the absence of pests and diseases. Reasons for quality loss are numerous. The quality of ornamental crops is realized in field or greenhouse by selecting the best genotype of the species of interest and the best cultivation conditions. Environmental conditions during preharvest such as temperature, relative humidity, light intensity, quality, and periodicity, are essential for crop physiology and influence quality and yield. The combination genotype and environment can be used to optimize to grow the best quality possible.

After harvest, the quality cannot be improved but only preserved. Postharvest storage conditions and treatments can be optimized to preserve quality; long vase life of cut flowers, and post-production life of potted plants at the consumer. This Research Topic collects reviews and research papers that cover quality, production, and distribution chain of ornamental crops. It is aimed to bring together research on innovation, technology, and sustainability of ornamental plant production, in order to stimulate the development of ornamental crop research, particularly focused on cut and potted flowers and foliage. In the 20 articles published, both reviews and experimental, the most recent advances on ornamental plants as biotechnology, physiology, nutrition, propagation, and pre- and postharvest treatments are highlighted.

## Quality aspects of ornamental crops

The aesthetic value of ornamental crops, combined with expected postharvest life defines the acceptance on the market. The first, is largely depended on shape, color, fragrance, and uniformity of blooming. Some of the articles are studies on the improvement of quality. Because humans cannot distinguish colors equally, a new digital image methodology—FloCIA (Flower Color Image Analysis) software, was developed for color evaluation in sunflower ray floret. The main advantage is the high accuracy of color category matching and the concise information that may be applied for color evaluation, mainly by breeders and traders (Zoric et al.). Fragrance emission was shown to be influenced by light and temperature during postharvest conditions. The quality of the scent is affected by light and temperature, indicating the importance of adequate pre-harvest conditions. For narcissus, 15°-5°C thermoperiod and a 12:12 Light Dark photoperiod provide the richest scent profile production. This information can be used to optimize or manipulate fragrance, which is good for consumers, and could also be interesting for the perfume industry (Terry et al.).

## Genotype and molecular biology strategies

Although the acceptance of transgenic crops is not widespread, the acceptance in ornamental crops is considered higher (Chandler and Sanchez, 2012). The unintentional release of GM petunia which could produce an orange color not previously seen in the genus demonstrates the possibilities, as well as the absence of long-term consequences (Bashandy and Teeri, 2017; Boutigny et al., 2020). A biotechnological study of genes related to a compact phenotype was carried out in *Kalanchoe blossfeldiana*. The over expression of the genes collectively responsible for hairy root or Ri phenotype resulted in a desired more compact plant morphology, and reduced ethylene sensitivity, resulting in increased postharvest longevity (Trevenzoli et al.). Another interesting approach is the use of interspecies hybrids to create new sources for breeding targets. The results obtained for Pelargonium may consist in a start point to study the genetics of organelle management and expression, concerning in an evolutionary context and/or genes involved in these processes studies (Breman et al.).

The processes of divergence and speciation, associated with the role of geography and climate changes, can be visualized with evolutionary research. Understanding these processes allows for estimating the genetic structure and demographic history of closely related species. A study with *Rhododendron dauricum* and *R. mucronulatum* was performed and showed the genetic differentiation and a clustered monophyletic group when SLAF data was used but not in cpDNA. The evolutionary process of these species was a consequence of an interspecific gene flow, and the divergence was maintained by natural selection (Yang et al.).

In woody plants native to temperate and boreal regions, bud dormancy and cold acclimation are strictly regulated induced by shortening daylength and low temperature. Dehydration is essential to decrease free water that could lead to mechanical stress caused by extracellular ice formation. Dehydrins are a subset of cold induced genes, involved with the plant protection against freezing. They also interact with carbohydrates, which are associated with cold hardiness. Together they may help the plant improve cold hardiness. During cold acclimation of roses cultivars, a *Dehydrin* (*RhDHN5*) was upregulated, but the stem water content remained stable. Differences in carbohydrate metabolism and related genes, could not be attributed to cold hardiness, indicating different cold adaptation strategies (Ouyang et al.).

## Preharvest

Proper cultivation is essential for yield and quality. Many (ornamental) plants are produced from stock plants by planting shoot tip cuttings that form adventitious roots. The review article from Druege summarizes the main processes involved in vegetative propagation by rooting of shoot tip cuttings, used for many ornamental plant species. Besides the influence of environmental factors and plant genotype, the author emphasizes the bottlenecks, the role of auxins and cytokinins, C, and N surplus and utilization, strigolactones, wound-induced accumulation of ethylene, and jasmonic acid, and the relation with the vitality of cuttings or insufficient adventitious root formation.

The author proposes a targeted approach to analyse and group plant species and cultivars according to potential bottlenecks, and proposes to combine this with the machine learning inspired concept of the “plant perceptron” (Scheres and van der Putten, 2017). Knowing these factors and roles may allow for improving propagation protocols, maximizing the plant genetic endogenous potential, and contributing to demand for sustainability (Druege).

Concerning advances in plant *in vitro* propagation, a methodology for direct somatic embryogenesis and organogenesis of clematis cultivars is presented, studying the effect of plant growth regulators by using several concentrations. In the study performed, the process was affected by the light intensity and temperature, besides it was observed that some cultivars presented a higher frequency of secondary somatic embryogenesis. Surprisingly, the response from different cultivars was quite similar. In other species, often large differences in shoot formation exist between genotypes. The results show an important advance, since it is the first time that the studied clematis cultivars have been shown to possess high morphogenic capacity due to a combination of methods applied during micropropagation (Mitrofanova et al.).

For plant production stages, beneficial bacteria may play an important role by acting as biostimulant for avoiding water-deficit stress in ornamental plants cultivated in the greenhouse. Bacteria were isolated from the rhizosphere of water stressed greenhouse ornamentals: coleus, petunia, geranium, vinca, and zinnia. Ten isolates were tested on petunia and geranium during discontinued irrigation. The combination of *in vitro* and greenhouse experiments led to the identification of Pseudomonas strains that can increase tolerance to and recovery from water stress (Nordstedt et al.).

In another approach, animal-based protein hydrolysate (PH) was tested for biostimulant properties. When applied as a foliar spray in petunia, contributed to achieve extra-grade plants, by improved net photosynthesis and stomatal conductance. This is a promising result since improving the metabolism, may turn nutrients absorption more efficient, leading to sustainable production practices (Cristiano and De Lucia).

During potted roses production, pinching is a technique that is used to stimulate branching. However, it can lead to leaves to turn yellow and abscise. The same problem occurs in plants under low light conditions, as in home and offices. To prevent this, the application of the cytokinin thidiazuron (TDZ) may be used, resulting in plants with shorter and thicker stems. The study performed by Çelikel et al. indicates the antagonistic effect of TDZ (cytokinin) and GA3 in potted rose in the control of stem maturation and elongation. It was demonstrated that TDZ regulates shoot elongation and stem enlargement through the modulation of bioactive GA biosynthesis. The application of both, Ga3 and TDZ resulted in normal elongation growth, maintaining the compactness, and being an alternative for plant height control.

Postharvest light has shown promise for the preservation of quality in postharvest, the practical application of light during postharvest is challenging because packing material (boxes etc.), as well as the plant products themselves potentially (partially) blocks the light to reach all plant tissues. An interesting approach discussed the effectiveness of external light stimuli on pre and postharvest, for improving the quality of cut flowers. Also, it is highlighted future research focus, considering the understanding of how light stimuli affect several features of cut flowers and investigating the reaction among species and cultivars (Horibe). Recent work has indicated that by applying specific treatments during the cultivation, quality can be improved (Affandi et al., 2020, 2022; Min et al., 2021). The results of the study by Terfa et al. show that increased blue light during preharvest improved stomatal function under high RH conditions. Ornamentals grown under high RH often suffer from stomatal malfunction leading to reduced vase life (Arve et al., 2013; Aliniaeifard and van Meeteren, 2014; Schouten et al., 2018). This was shown to be connected to increased [ABA]. The degradation of ABA-GE by β-glucosidase steers the diurnal ABA pool turnover, and this activity was shown to be induced by Blue LED light. The B-light during the day led to increased β-glucosidase activity during the night that led to ABA release. This preharvest B-light could be used to improve postharvest water balance of roses.

## Improved production chains

Quality can be preserved during the distribution chain by controlling the post-production conditions (Manfredini et al., 2017; Sales et al., 2021). Cut flowers quality can be preserved after harvest by lowering the metabolism. Immediately after harvest the temperature must be lowered for reducing respiration, ethylene production, and water loss. Recently, it has been reported the importance of light quality during the storage of cut flowers. In chilling sensitive Anthurium, it was shown that the light spectrum could reduce chilling injury symptoms. Cut anthurium flowers showed the longest vase life if stored under red light and the shortest vase life was observed in cut spathes exposed to blue light (Aliniaeifard et al.). On the contrary, for carnation cut flowers, blue light exposure improves vase life, by inducing the antioxidant defense system in petals (Aalifar et al.).

In cut flower conservation after harvest, the use of auxins is not very common. There are not many studies that explore the use. In Red Cestrum, pulse treatments of auxin demonstrated that addition of auxin can reduce floret bud abscission, and that the efficacy was improved by raising the solution pH since this condition contributes to the auxins acropetally transport (Abebie et al.).

The use of putrescine was investigated as a new strategy for cut foliage conservation after harvest, showing to be effective to alleviate senescence. Spraying putrescine on foliage may prevent membrane impairment and injury, besides affecting plant metabolism, and delaying senescence (Qu et al.). A promising nutrient for postharvest is selenium and its role on the longevity of ethylene-sensitive flowers was discussed in a review indicating a positive alternative, commercially viable and environmentally friendly (Costa et al.).

## Future and outlook

The potential new products may be found by observations of the efficacy in other species, as well as in animals and humans. For example, in the study developed by Li et al., they found that Magnesium hydride (MgH_2_) which is a solid-state hydrogen source with high storage capacity (7.6 wt%), is a potential product for postharvest. Initially evaluated in medicine, the effectiveness was also observed in plants, analyzing cur carnation flowers. The results showed that MgH_2_-supplying H_2_ could prolong the vase life *via* H_2_S signaling. Also, this indicates that future studies may consider a possible application of hydrogen-releasing not only for postharvest but also for production (Li et al.).

The use of mathematics, machine learning information-processing system, connecting with some recent concepts as plant perceptron were presented by Druege as application approaches for plant studies. This may be in connection with early plant responses, as it was demonstrated for cutting root formation concerning the role of plant hormones JA, ET or IAA and the Aux/IAA-ARF modules (Druege).

The mechanisms and key factors in the plant-microorganism-abiotic environment are indicated as future subject for research on plant propagation (Druege).

A relatively new topic is to use the light spectrum, intensity, and perhaps period to improve quality. This can be done during pre and postharvest as discussed above. Although there are many studies in all phases of plant life, the effects of different light spectra on post-harvest performance of cut flowers are largely unknown. To advance in this knowledge, the use of light emitting diodes (LEDs), allows studying the main light spectra Red (R) and blue (B) wavelengths on plants. The low energy use and lower temperature released by LED modules have improved possibilities to apply them, as well as the reduced costs. In post-harvest phase, an enhanced of the activity of antioxidant systems may occur in consequence of the light spectra, affecting the longevity, but some detailed studies are still required. In this way, these results show important contributions for flower post-harvest.

In the end, all the publications of this Research Topic contributed with outstanding information for the ornamental plant area.

## Author contributions

All authors listed, have made substantial, direct, and intellectual contributions to the topic, and approved it for publication.

## Conflict of interest

The authors declare that the research was conducted in the absence of any commercial or financial relationships that could be construed as a potential conflict of interest.

## Publisher's note

All claims expressed in this article are solely those of the authors and do not necessarily represent those of their affiliated organizations, or those of the publisher, the editors and the reviewers. Any product that may be evaluated in this article, or claim that may be made by its manufacturer, is not guaranteed or endorsed by the publisher.
